# Clinical Lecture on Psoriasis

**Published:** 1894-10

**Authors:** William S. Gottheil

**Affiliations:** Dermatologist to the Lebanon Hospital, the Northwestern and the German West-Side Dispensaries, New York


### CLINICAL LECTURE ON PSORIASIS.

By WILLIAM S. GOTTHEIL, M.D.,

Dermatologist to the Lebanon Hospital, the Northwestern and the German West-Side Dispensaries, New York.

Gentlemen:—The patients that I show you to-day are classical examples of a common disease, and are, on that account perhaps, more worthy of our attention than those rarer affections that but very seldom come to the notice of the general practitioner. And they will serve me as a text in calling your attention to certain new and very eligible forms of treatment that have been developed in the last few years, and which have largely superseded the older methods.

The first patient is an excellent example of a general guttate psoriasis, psoriasis universalis, in a female thirty-three years of age. She has had the malady, to her own recollection, ever since her fifth year, the usual history of these cases; though it does occur *de novo* even in advanced age, and the defected memory and carelessness of our dispensary cases often lead them to claim that the present is their first attack. It has been constantly present, in some degree, ever since the patient can recollect; at times almost disappearing, and then, under influences that we are ignorant of, advancing and spreading over the body until it occupies areas as extensive as that which you see affected at the present time. Her entire body is covered with white, scaly spots, looking very much as if some molten waxy material had been liberally sprinkled on it with a large brush. Each such spot consists of a heaped up mass of silvery epidermic scales, which can be readily removed with the finger nail, leaving a reddish, slightly elevated papule behind, at points of which the torn tops of the papillæ of the skin show as minute bleeding points. The scales are lamellæ of fused epidermic cells, and their peculiar silvery appearance is due to presence of air between them.

The entire surface of the body is sprinkled with these guttæ; but in certain localities, and more especially on the flexor surfaces of the joints of the extremities, they are most abundant, and form more or less continuous scaly masses, with but little healthy skin between them. So abundant is this scaling that the patient scatters a cloud of minute lamellæ around her as she moves when stripped; and several large handfuls can be gotten from her clothing. The epidermic proliferation is quite rapid in these cases; but it is only on parts not often washed that it occurs to so great an extent as you see. On the face and hands, where soap and water have not been quite so sparingly employed, there are no scales at all; only the low reddish papules mark the existence of the disease. It is important to note this fact; for in some cases, when the disease is not extensive, the patients have removed all the scales before they come, and the apparent absence of so characteristic a symptom may lead to an error in diagnosis. The scalp is covered with more or less confluent psoriatic patches, but the palms and soles are free.

The second case is a male about the same age, with a very different but just as characteristic disease appearance. Only the knees and elbows are affected. Each of these surfaces, where the skin is naturally thicker and rougher than on other portions of the body, shows a more or less extensive infiltrated patch, with apparently but little scaling; but scraping reveals the characteristic lamellæ. Here also the condition has existed for many years; the scaly infiltrated patches disappear at times, especially during the hot weather; but they always reappear during the winter.

Both patients are evidently in good health; in fact, most psoriatic patients are robust, even when the disease is very extensive. Its cause is absolutely unknown. Heredity certainly plays no part in it. It may be of parasitic origin; but no microbe has been found. The epidermophyton described by Langer is certainly not the etiological factor.

It is to the treatment of these cases, however, that I would call your especial attention. Internal medication is of the greatest importance, especially in cases so extensive as our first one. Arsenic, so little employed by the dermatologist, is undoubtedly of use here, German opinion to the contrary notwithstanding. But it must be taken regularly, and in large doses, and for a long time. It is therefore given in the pill form. Ichthyol is also beneficial; and we will put both patients on a combination of the two, using a modification of the famous "Asiatic Pill," which is a favorite formula of mine:

℞ Ammon. sulph ichthyolat.......................................ʒ ii.

Acid. arseniosi.......................................................gr. iii.

Pulv. pip. nig........................................................℈ iii.

Pulv. glyce. rad.....................................................℈ iii.

M. Ft. pil. No. 90.

One of these is to be taken three times daily, after meals. The amount of arsenic may be gradually increase until a maximum dose of one-twentieth or one-fifteenth grain is attained.

Local treatment, however, is of even greater importance than internal medication. It is essential in all cases, and is especially important when the face and hands are affected with the disease. The deformity must be removed as rapidly as possible.

Our local treatment will differ in the two cases. In the first and general one, it should be systematic and thorough, and it may be summarized as follows:

1. Daily general bath of hot water and green soap. The scales must be entirely cleaned off from the surface of the body, to permit the appliance of topical remedies.

2 After leaving the bath, paint each spot with:

℞ Ol. rusci., or ol. cadini........................................ʒ ii.

Spirit. vini.

Etheris.............................................................āā ʒ iv.

Spirit. lavandulæ..................................................gtt. x.

3. Return to the bath, and remain there half an hour.

4. After drying, paint each spot with the following:

℞ Arthrarobin, or chrysarobin.................................1 part.
Liquor gutta percha, or,
Flexible collodion.................................................10 parts.

Arthrarobin is not quite so effective as chrysarobin; but it is safer. It may be employed over the entire body, whilst chrysophanic acid must not be used on the face or hands; not only on account of the very dark staining of the skin that it causes, but also on account of the likelihood of its causing the disagreeable and even dangerous "chrysarobin conjunctivitis." If we decide to use it, the ungt. hydrargyri ammoniati must be employed on the face and hands.

By this means the inunction of the whole body with disagreeable ointments, the use of cloths and bandages, and all the nasty paraphernalia of the regular ointment treatment, are avoided ; and the clothing, inevitably ruined in the older methods, is in no way harmed. The evaporation of the etherial and alcoholic vehicles of the remedies leaves them in a thin and hard layer on the skin ; and their penetration in these solutions is at least as great as when suspended in the ordinary fatty vehicles.

The local treatment of the second case is more simple. We now possess in the unguenta extensa, collemplastra, and the plaster-mulls, a variety of very eligible preparations, which are really ointments spread on plaster, and so combined with the basis that they can be used and applied like ordinary rubber plaster. We simply take some of the ten per cent. chrysarobin plaster-mull, cut a piece to accurately cover the psoriatic spots, and apply them. They fit accurately to the parts, need no cloths or bandages to hold them in place, do not soil the clothing, and, above all, limit the action of the remedy exactly to the diseased area. We will direct the patient to renew these plasters daily until the patches are cured.

Shall we succeed in curing our cases ? Yes, for the time being. Every spot of psoriasis will disappear from the skin, but others will come back in time to take their places.

25 *West 53d Street, New York City.*

---

### PEROXIDE OF HYDROGEN IN STOMATITIS.

Boennecken prefers this solution in strength from two to five per cent. to solutions of potassium chlorate or permanganate, especially in febrile and wasting diseases. It destroys fetor in a short space of time, it is non-irritant, and if used continuously the buccal mucous membrane will show decided improvement in twenty-four hours.

In discussing the paper, Leo stated that his results were also good with the drug. Walters praised its effect in five to ten per cent. solutions in mercurial stomatitis; but Bing regarded potassium chlorate as equally effective.—DR. JOHNSTON, in *Journal of Cutaneous and Genito-Urinary Diseases*, June, 1894.

